# A tutorial on Bayesian single-test reliability analysis with JASP

**DOI:** 10.3758/s13428-021-01778-0

**Published:** 2022-05-17

**Authors:** Julius M. Pfadt, Don van den Bergh, Klaas Sijtsma, Eric-Jan Wagenmakers

**Affiliations:** 1 Department of Psychological Research Methods, University of Ulm, Ulm, Germany; 2 Department of Psychological Methods, University of Amsterdam, Amsterdam, The Netherlands; 3 Department of Methodology and Statistics, Tilburg University, Tilburg, The Netherlands

**Keywords:** Credible interval, McDonald’s omega

## Abstract

The current practice of reliability analysis is both uniform and troublesome: most reports consider only Cronbach’s *α*, and almost all reports focus exclusively on a point estimate, disregarding the impact of sampling error. In an attempt to improve the status quo we have implemented Bayesian estimation routines for five popular single-test reliability coefficients in the open-source statistical software program JASP. Using JASP, researchers can easily obtain Bayesian credible intervals to indicate a range of plausible values and thereby quantify the precision of the point estimate. In addition, researchers may use the posterior distribution of the reliability coefficients to address practically relevant questions such as “What is the probability that the reliability of my test is larger than a threshold value of .80?”. In this tutorial article, we outline how to conduct a Bayesian reliability analysis in JASP and correctly interpret the results. By making available a computationally complex procedure in an easy-to-use software package, we hope to motivate researchers to include uncertainty estimates whenever reporting the results of a single-test reliability analysis.

“There is no excuse whatever for omitting to give a properly determined standard error (...) All statisticians will agree with me here (...)” Harold Jeffreys (1961, p. 410)

The concept of reliability plays a key role in psychological research involving tests and questionnaires. In general, reliability quantifies the degree to which a measurement instrument provides similar results in repeated application. For instance, before buying a bathroom scale you may decide to try it out several times in quick succession. If the returned weights are equal, the scale is perfectly reliable; if the returned weights vary substantially, the scale is unreliable and any individual result ought to be viewed with caution. Although in this example only one individual is measured and, classically, reliability is defined for a group of individuals, the underlying idea remains the same: The similarity of repeated measurements (of a group or individual) indicates the degree to which the measurement is reliable.

In most applications in psychology, memory effects prohibit the use of repeated administrations of the same test. Instead one may consider parallel tests – different versions of the same test that are interchangeable except for random measurement error. Reliability defined as the correlation between two parallel tests mathematically equals the proportion of test score variance that is not due to random measurement error (Lord & Novick, 1968). When parallel tests are infeasible, impractical, or unavailable, researchers have to try and disentangle true score variance from the overall test score variance using the data from a single test administration (for more information, see, e.g., Sijtsma & Van der Ark, 2021).

Single-test reliability can be estimated by several different coefficients, the dominant one being Cronbach’s *α* (Cronbach, 1951). Coefficient *α* is a lower bound to the reliability, and is based on the covariance between the questionnaire items. When the underlying scale is unidimensional and when every item captures the true score equally well, then *α* equals reliability (Lord & Novick, 1968). Under more general conditions, coefficient *α* is considered as a lower bound on reliability (e.g., Dunn et al., 2014; Sijtsma, 2009).

Despite ongoing methodological debate about the pros and cons of the different single-test reliability coefficients (e.g., McNeish, 2018), scientific practice manifests an approach to reliability analysis that is both near-unanimous and troubling. Point estimates of reliability coefficients are virtually never accompanied by any measure of precision. For instance, Flake et al., (2017) encountered uncertainty intervals for fewer than 5 out of 301 coefficients (personal communication, August 3, 2020); Moshagen et al., (2019) did not encounter any uncertainty intervals for 549 coefficients (personal communication, August 3, 2020); similarly, Oosterwijk et al., (2019) did not encounter any uncertainty intervals for 1,024 coefficients. We suspect that the lack of uncertainty reporting is partly due to a common misunderstanding: Since reliability is a quantification of measurement error itself, researchers fail to view reliability as a parameter that is affected by measurement error and thereby necessitates an uncertainty estimate.

As a running example throughout this manuscript we use data from the Altman Self-Rating Mania Scale (ASRM) which was used by Nicolai and Moshagen (2018) as a possible control variable in a multiple regression model that quantified the association between pathological buying and the judgement of elapsed time. The ASRM consists of five 0-4 Likert-scored response items and was filled out by 78 participants. Standard reporting practice is to communicate as a measure of single-test reliability (a) only Cronbach’s *α*; (b) only the frequentist point-estimate, which for the ASRM data equals $\hat {\alpha }=.79$. Without an associated uncertainty interval, this point estimate is impossible to interpret.

In this manuscript we present a Bayesian framework which allows researchers to obtain Bayesian uncertainty intervals (generally known as *credible intervals*) for five different single-test reliability coefficients. More generally, the methodology discussed below allows researchers to obtain entire posterior distributions for single-test reliability coefficients. A posterior distribution represents the relative plausibility of the coefficient values after the observed data have been taken into account. One obtains a posterior distribution by updating a prior distribution by means of the likelihood of the data. The prior distribution represents the relative plausibility of the parameter values before the data have been observed.

For the ASRM data, a default Bayesian analysis for Cronbach’s *α* allows a researcher to draw the following conclusions:

1. The posterior mean for Cronbach’s *α* equals 0.785. This provides a Bayesian point estimate.
2. A 95% Bayesian credible interval for Cronbach’s *α* ranges from .706 to .852.^1^ In other words, there is a 95% probability that Cronbach’s *α* lies in the interval [.706,.852]. This Bayesian credible interval is analogous to the frequentist confidence interval, which is often numerically similar (Pfadt et al., 2021).
3. Let the interval between *α* = .70 and *α* = .90 be of particular interest. This interval contains 97.8% of the posterior mass; in other words, there is a 97.8% probability that Cronbach’s *α* is larger than .70 and smaller than .90. This Bayesian interval estimate is fundamentally unavailable in frequentist methodology (e.g., Pratt et al., 1995; Wagenmakers et al., 2018). Frequentist methods can produce (1 − *α*)% confidence intervals (*α* being the significance level), but they cannot produce the confidence that is associated with any specific interval (Morey et al., 2016).

We assume that readers of this tutorial are sympathetic to conducting a reliability analysis in the Bayesian instead of the frequentist framework (e.g., Vandekerckhove et al., 2018; Wagenmakers et al., 2018). For a comprehensive tutorial on a frequentist reliability analysis in R, including confidence intervals, see Revelle and Condon (2019).

Researchers interested in applying a Bayesian single-test reliability analysis are confronted with three major challenges: (1) How to develop and implement a statistical procedure that produces the desired posterior distributions; (2) How to execute a Bayesian reliability analysis in available software; and (3) How to interpret the results correctly.

The first challenge was overcome by Padilla and Zhang (2011) and Pfadt et al., (2021). Padilla and Zhang introduced a Bayesian version of Cronbach’s *α* and Pfadt et al., described Bayesian versions of three additional reliability coefficients: Guttman’s *λ*_2_ (Guttman, 1945), the greatest lower bound (glb; Woodhouse & Jackson, 1977), and McDonald’s *ω* (McDonald, 1970; 1999). Readers interested in the methodological background of the Bayesian coefficients may consult Padilla and Zhang (2011) and Pfadt et al., (2021). All formerly mentioned reliability coefficients have been implemented in an R-package and in JASP, an open-source statistical software program with an intuitive graphical user interface.^2^ In this tutorial, we describe how to overcome the second and third challenges: we outline how to conduct a Bayesian reliability analysis in JASP and how to correctly interpret the results. The ?? shows how to conduct the analysis in R.

JASP is a statistical software program with a graphical user interface (GUI). It is aimed at researchers not versed in programming languages such as R. Other GUI programs, namely SPSS (v25), Stata (v16.1), Statistica (v13), Minitab (v19.2), and JMP Pro (v15), offer limited functionality to estimate uncertainty in a reliability analysis. Only JMP Pro provides bootstrapping methods to obtain a confidence interval for Cronbach’s *α*, whereas SPSS and Stata have workarounds; none of these programs offers a credible interval or a posterior distribution.

Although in this tutorial conducting a Bayesian reliability analysis will seem relatively straightforward, we argue that a comprehensive reliability analysis is rather complex. In particular, one should first determine the reliability approach one wishes to use, classical test theory, factor analysis, or generalizability theory; one should check if the assumptions of the chosen approach are met and then select the reliability coefficients accordingly. For more guidance on the measurement models underlying different reliability coefficients, we refer readers to Flora (2020), McNeish (2018), Savalei and Reise (2019), and Sijtsma (2009), or, more generally, Sijtsma and Van der Ark (2021, Chapter 2).

## Conducting a Bayesian single-test reliability analysis in JASP

We will conduct a Bayesian reliability analysis for the ASRM example in JASP. The ASRM data file (example_asrm.csv) and the associated article are available in an OSF-repository at https://osf.io/s4qr5/.^3^

**Fig. 1:**
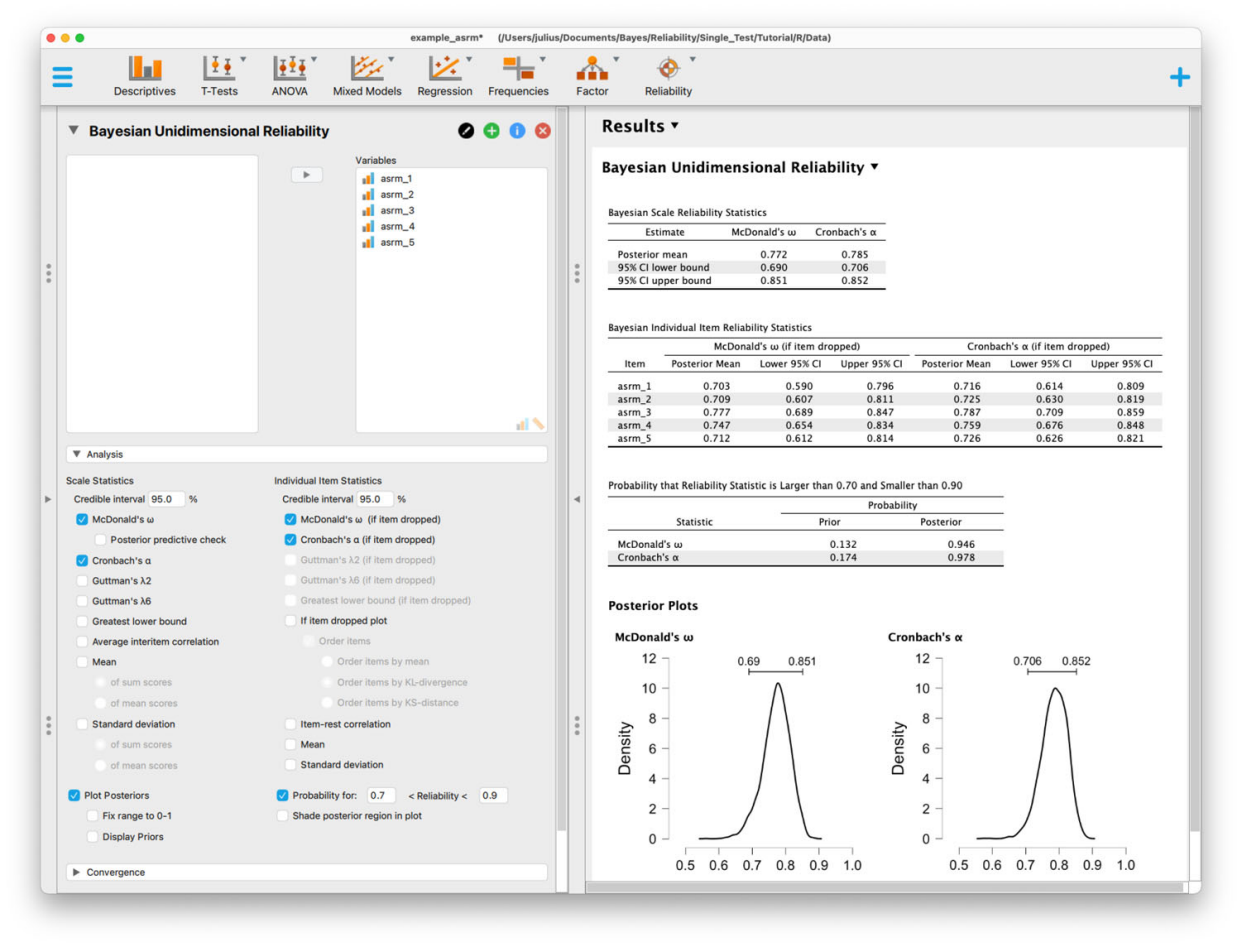
Screenshot of the Bayesian reliability module in JASP as applied to the ASRM data. The options specified in the left analysis input panel yield corresponding results displayed in the right output panel

First we open the example_asrm.csv file in JASP. After the data have been loaded we click on the blue “+” symbol in the top right corner of the JASP window in order to access the module list. In the module list we tick “Reliability”; the reliability module is now activated, and the matching icon appears on the ribbon above, next to the other analyses. Clicking the icon unfolds a menu from which we select, under “Bayesian”, the option “Unidimensional Reliability”. The left panel shown in Fig. 1 provides a screenshot of some of the resulting analysis input options.

### Basic analysis

In order to initiate an analysis we select all five Likert items from the input panel and move them to the “Variables” pane. The resulting analysis output – a point estimate and 95% credible interval for McDonald’s *ω*– is then displayed as a table in the output panel (cf. Fig. 1). Unfolding the “Analysis” tab underneath the variables pane confirms that McDonald’s *ω* has been pre-selected as the default choice.

**Fig. 2:**
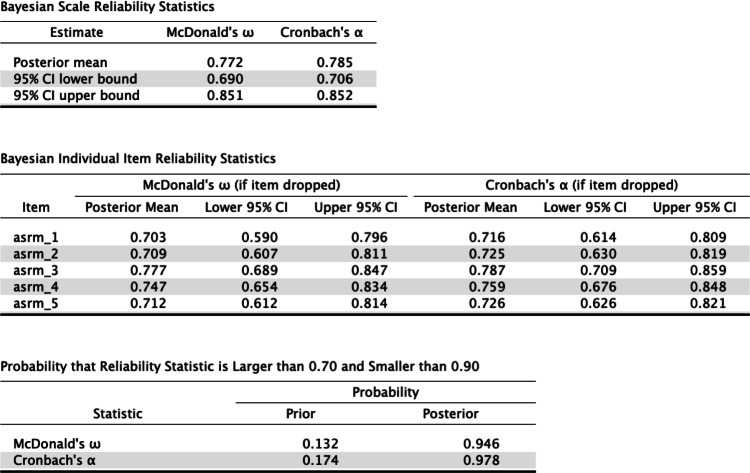
JASP output tables. Top table: point estimates and credible intervals for McDonald’s *ω*, Cronbach’s *α*. Middle table: the “if item dropped” statistics. Bottom table: the prior and posterior probability that a reliability coefficient lies between two user-defined cutoffs

JASP offers five estimators of single-test reliability: McDonald’s *ω*, Cronbach’s *α*, Guttman’s *λ*_2_, Guttman’s *λ*_6_, and the Greatest lower bound (glb). We retain McDonald’s *ω* and tick Cronbach’s *α*.^4^ The table in the output panel is then updated to include the point estimate and 95% credible interval for Cronbach’s *α* (see the top table in Fig. 2). The analysis is based on samples from the posterior distribution, and therefore the estimates may vary slightly when rerun.^5^

The results may be reported as follows: “For McDonald’s *ω*, the posterior mean equals .772 and the 95% credible interval ranges from .690 to .851; for Cronbach’s *α*, the posterior mean equals .785 and the 95% HPD credible interval ranges from .706 to .852. The probability that McDonald’s *ω* lies between .690 and .851 is 95%; the probability that Cronbach’s *α* lies between .706 and .852 is 95%.”

In addition, researchers may be interested in the posterior probability that a particular coefficient lies in a specific interval of interest or exceeds a certain value. This interval of interest can be defined by ticking the box “Probability for:” and specifying the lower and upper limit. The corresponding prior and posterior probabilities are then displayed in a separate table (see the bottom table in Fig. 2). For the ASRM data, the data have increased the probability that McDonald’s *ω* falls in the .70 − .90 interval from .132 (i.e., the prior probability) to .946 (i.e., the posterior probability).

### Displaying posterior distributions

**Fig. 3:**
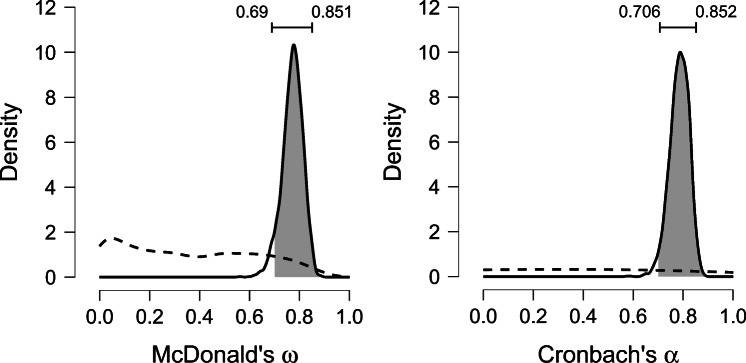
Prior and posterior distributions for McDonald’s and *ω* and Cronbach’s *α*. The dashed and solid lines correspond to the prior and posterior distribution, respectively. The 95% HPD credible interval is displayed on top, and the shading marks the interval specified in the “Probability for:” input fields (here: .70 and .90). Figures from JASP

Ticking the box “Plot Posteriors” produces a plot of the posterior distributions of the reliability coefficients. The HPD credible interval is indicated by a horizontal bar above the density curve. Ticking “Display Priors” adds the prior distributions; ticking “Shade posterior region in plot” visualizes the interval of interest. The resulting output for the ASRM data is shown in Fig. 3. The posterior distribution shows the relative plausibility of the parameter values and the informativeness of the data. Narrow posterior distributions indicate that only a small subset of values are plausible, and that the estimation has been relatively precise. This information is also contained in the posterior mean and the 95% credible interval, but ultimately these numbers only summarize the complete posterior distribution, the proper interpretation of which usually benefits from a visual inspection. For further information on probability distributions and their interpretation, see, for example, (Kruschke, 2015, chapter 4).

### If-item-dropped statistics

The reliability module includes common item statistics, such as the item-rest correlation (also known as the corrected item-total correlation) and the values of reliability coefficients when an item is dropped. Selecting McDonald’s *ω* and Cronbach’s *α* “(if item dropped)” brings up the middle table in Fig. 2. The deletion of item 3 improves reliability by a minuscule amount (i.e., the posterior mean for McDonald’s *ω* increases from .772 to .777; Cronbach’s *α* increases from .785 to .787). Deleting any of the other items leads to a decrease in reliability.

**Fig. 4:**
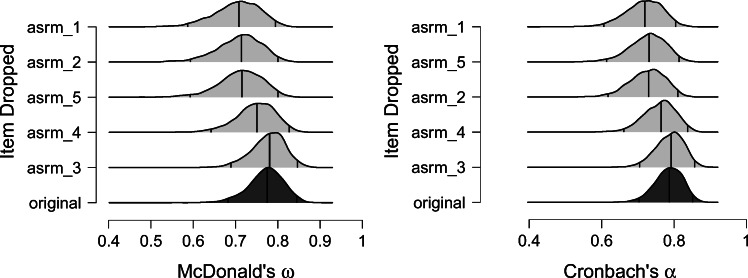
The ordered posterior densities when items are dropped. The bottom density shows the posterior with the original number of items. Going from bottom to top, the difference to the original density increases. The ordering is based on the KL-divergence. Figures from JASP

The consequences of dropping an item may be visualized by clicking on “If item dropped plot” (see Fig. 4). The resulting posterior distributions can be ordered in different ways: (a) by the difference between the posterior means; (b) by the Kullback-Leibler (KL) divergence (Kullback & Leibler, 1951); and (c) by the Kolmogorov-Smirnov (KS) distance (Kolmogorov, 1933; Smirnov, 1939). For further information about the metrics to measure the difference between distributions, see, for example, Gibbs and Su (2002). Figure 4 orders the posterior distributions by KL-divergence. This figure shows that the reliability coefficients decrease the most when item 1 is deleted (i.e., the posterior distribution displayed in the top row). Deleting item 3 does not change the posterior distributions in a meaningful way. We note that the deletion of an item should never be based purely on statistical information but preferably involve theoretical considerations. We believe the display of the posterior distributions for this purpose (see Fig. 4) may prevent researchers from rash decisions by visualizing the loss in information that would arise from deleting an item. Usually for unidimensional data, the deletion of an item will result in a wider posterior distribution, that is, more uncertainty around the point estimate.

### Assessing MCMC convergence

The reliability analysis in JASP uses the R-package Bayesrel to compute the reliability estimates. The R-package obtains posterior samples of the reliability coefficients by means of Markov chain Monte Carlo sampling (MCMC; e.g., Gilks et al., 1995).^6^ For further information about MCMC sampling and convergence, see, for example, Van Ravenzwaaij et al., (2018). In JASP, the details of the MCMC algorithm can be controlled through options available under the menu “Convergence”. We briefly summarize the rationale for these options below.

The MCMC sampling algorithm starts with random parameter values and then converges to the posterior distribution as more and more samples are drawn. In the initial phase of this process (known as “burn-in”) the sampled parameter values still depend on their starting values and are therefore not representative of the posterior distribution. Such burn-in samples should be discarded. In order to help assess whether the MCMC sampling has converged to the posterior distribution, it is customary to run the algorithm several times with different starting values; these different runs are known as *chains*. When the different chains have converged to the posterior, they should “mix” well. The extent to which the chains are mixing can be quantified by the “R-hat” statistic (Gelman and Rubin, 1992) which compares the within-chain variance to the between-chain variance; for chains that mix well the R-hat statistic is close to 1. An R-hat statistic larger than 1.1 is considered problematic (Gelman et al., 2014, chapter 11.5). In our example, R-hat is 1.000 and 1.003 for *ω* and *α* respectively (see the top table in Fig. 2).

As the name “chain” suggests, consecutive MCMC draws are usually correlated. High levels of autocorrelation indicate that the sampling process moves slowly through the posterior distribution, and this limits the efficiency with which the posterior can be approximated. A common method to reduce autocorrelation is known as “thinning”. For instance, a thinning interval of 2 means that every other value from the original chain is discarded. In order to assess convergence it is usually helpful to display the successive values of the chains; these displays are known as “traceplots”.

**Fig. 5:**
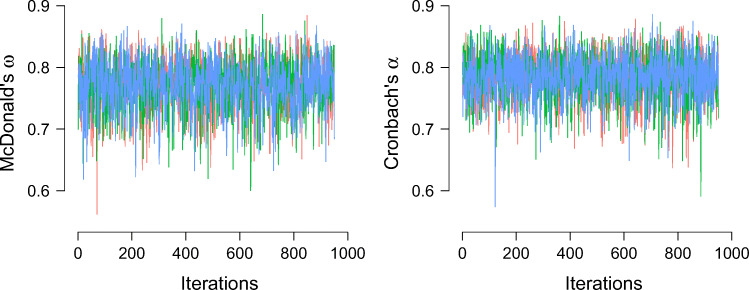
Traceplots of the MCMC samples for reliability coefficients McDonalds *ω* (left panel) and Cronbach’s *α* (right panel) applied to the ASRM-data with 1,000 iterations, a burn-in of 50, thinning interval of 1, and three chains (represented by different colors). Figures from JASP

In JASP, the options under the “Convergence” menu allow users to adjust the number of chains, the number of samples, the length of the burn-in, and the length of the thinning interval. In addition, users can obtain the R-hat statistic and inspect the traceplots. In our experience it is rarely necessary to change the default options. Figure 5 shows the traceplots for the ASRM-data. The traceplots show that the sampled values do not differ systematically depending on the chain or depending on the number of iterations, suggesting convergence to the posterior distribution.

### Prior distributions

By unfolding the tab “Priors” one may adjust the hyperparameters of the prior distributions for the reliability coefficients. The prior distribution on Cronbach’s *α* and other CTT-coefficients is induced by the prior distribution on the covariance matrix, which is an inverse Wishart distribution. The parameters of the prior inverse Wishart distribution are the identity matrix multiplied by a scalar (denoted “Scale”) as a scaling matrix and the degrees of freedom (denoted “Df”). The *Scale* value functions as a precision parameter and is by default set to 10^− 10^; the *Df* are by default set to the number of items *k* and are always at least as large as *k*.

Since McDonald’s *ω* is computed from the parameters of the single-factor model, its prior distribution is induced by the prior distributions on the single-factor model parameters. Specifically, the prior distributions are, by default, an inverse gamma distribution with shape *α* = 2 and scale *β* = 1 on the residual variances; a normal distribution centered on zero for the factor loadings and scores; and, on the variance of the latent variables an inverse Wishart distribution with the number of items *k* as a scaling matrix (more precisely, a scalar, since only one latent variable is specified) and *k* + 2 as the degrees of freedom. The choice of relatively uninformative priors for the factor model parameters results in a prior distribution of *ω* that is not uniform, but assigns less mass near values of 1 (see Fig. 3).

JASP offers control over the inverse gamma prior on the residual variances (“shape” and “scale” boxes) and the mean of the normal prior on the factor loadings (“mean” box). Among the prior parameters, the priors on the residual variances and the factor loadings are the most influential for the prior and posterior distribution of McDonald’s *ω*.

We consider the chosen prior parameters as relatively uninformative about the covariance matrix and the factor model. Users wishing to incorporate more prior knowledge into their analysis may adjust the prior parameters to better represent their assumptions. We advise to always compare the results from a more informative prior with the default (relatively uninformative) priors.

### Advanced options

#### Missing values

Unfolding the tab “Advanced Options”, we can treat missing values either with “Bayesian imputation” or “Exclude cases listwise”. For listwise deletion each row (participant) that contains at least one missing value is deleted from the data set in its entirety. When the data contain missing values and the user chooses Bayesian imputation, the Bayesian analysis will treat the missing data as to-be-estimated parameters. The missing values are sampled conditional on the remaining data and the sampled model parameters. This way we obtain a posterior distribution of each missing value (e.g., Schafer, 1999).

#### McDonald’s *ω* estimation

**Fig. 6:**
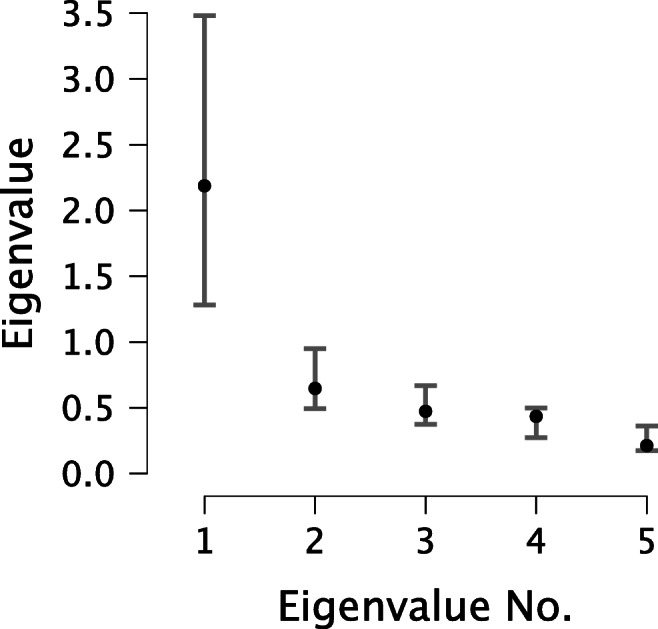
The posterior predictive check for the fit of the unidimensional factor model to the ASRM-data. The 95% intervals of the simulated eigenvalues from the model-implied covariance matrix are shown as grey lines, and the black dots represent the eigenvalues of the data covariance matrix. The fit appears satisfactory. Figure from JASP

McDonald’s *ω* is based on the unidimensional factor model and quantifies the general factor saturation when the unidimensional model fits.^7^ Post-hoc model fit can be checked in JASP by ticking the box “Posterior predictive check” (PPC; Gelman et al., 2014, chapter 6.3). The resulting figure shows how closely the data resemble the unidimensional factor model (see Fig. 6 for the ASRM-data). Specifically, the PPC-plot displays the eigenvalues of the data covariance matrix (black dots) together with 95% intervals (grey bars) based on eigenvalues simulated from the unidimensional model.^8^ In Fig. 6, all black dots fall inside of the intervals, suggesting that the unidimensional model provides a satisfactory fit to the observed data. We note that the PPC should only function as a post-hoc check to confirm that the unidimensional factor model fits the data, that is, McDonald’s *ω* is an appropriate reliability coefficient. One may obtain fit measures for the Bayesian single-factor model by checking the corresponding box “Fit measures”. These measures include Bayesian versions of the root mean square error of approximation (RMSEA), comparative fit index (CFI), Tucker-Lewis index (TLI), and a Bayesian version of the likelihood ratio (LR) test-statistic (Garnier-Villarreal & Jorgensen, 2020; Levy, 2011). For the ASRM-data these are: *BRMSEA* = 0.131, *p*(*BRMSEA* < .08) = 0.116; *BCFI* = .929, *p*(*BCFI* > .90) = .771; *BTLI* = .863, *p*(*BTLI* > .90) = .384; and *BLR* = 13.31. Interpreting the PPC-plot together with these fit values we can merely confirm mediocre fit of the single-factor model, and advise to treat McDonald’s *ω* with caution. Ideally, when using the factor analytic approach to reliability, researchers should determine the proper measurement model for the data before conducting a reliability analysis (see, e.g., Savalei & Reise, 2019).

Further, users can choose to display the standardized loadings of the single-factor model by checking the corresponding box. The resulting table displays the mean or median (see “Posterior point estimate”) of the standardized posterior factor loadings.

### Guidance on estimators

In light of the many critiques on Cronbach’s *α* (e.g., Cho, 2016; McNeish, 2018) and the advocacy of alternative estimators (Oosterwijk et al., 2016; Revelle & Zinbarg, 2009; Sijtsma, 2009), below we briefly mention some key properties of the various single-test reliability coefficients.

First, Cronbach’s *α* is a lower bound for reliability, meaning that it is an underestimate of the true reliability (Lord & Novick, 1968). When data are unidimensional, the degree of the underestimation is usually small (e.g., Dunn et al., 2014). Guttman’s *λ*_2_ has the same properties as *α* but is at least as large as *α* (Guttman, 1945; Oosterwijk et al., 2016). Guttman’s *λ*_6_ usually has a larger positive bias than *λ*_2_ with respect to its population value, and this bias increases with the number of items (Oosterwijk et al., 2016). In theory, the glb is the lower bound of choice for both unidimensional and multidimensional data (Oosterwijk et al., 2017; Sijtsma, 2009), but in practice the glb shows considerable positive bias and should only be reported for data sets with more than 1000 observations and fewer than 10 items (Ten Berge & Sočan, 2004).

McDonald’s *ω* is based on the unidimensional factor model and therefore can only approximate reliability when the factor model is an acceptable model for the data. In addition to the interpretation of *ω* as a measure of reliability, the coefficient also indicates how well a test measures a single factor (when the data are unidimensional).

Although unidimensionality is not an assumption for the derivation of the lower bound theorem for Cronbach’s *α* (the same is true for Guttman’s *λ*_2_ and *λ*_6_; Lord & Novick, 1968), the performance of the coefficient benefits from unidimensional data. Therefore, we urge researchers to make sure their data are unidimensional before estimating McDonald’s *ω*, Cronbach’s *α*, and Guttman’s *λ*_2_ both in the frequentist and the Bayesian framework.^9^ Whether data are unidimensional or not, the issue of whether the item set measures the intended attribute well is a validity issue. It cannot be settled by ascertaining unidimensionality and reliability.

## Concluding comments

Whenever researchers report a single-test coefficient of reliability they overwhelmingly resort to Cronbach’s *α* and they almost never accompany the point estimate by an uncertainty interval. This reporting routine is statistically sub-optimal, but existing software does not offer an easy alternative to researchers without programming expertise. To facilitate a more complete reporting practice we implemented five Bayesian reliability coefficients in JASP, an open-source statistics program with an intuitive graphical user interface.

In this tutorial paper we demonstrated how to conduct a Bayesian reliability analysis in JASP and how to interpret the results. With JASP, it is straightforward to obtain a posterior distribution for a reliability coefficient. This posterior distribution can then be interrogated in several ways: one may obtain a point estimate, a credible interval, and the probability that the coefficient falls within a specific interval of interest. One may also explore the change in the posterior distribution when an item is deleted, one may adjust the settings of the MCMC sampling algorithm, and one may check the extent to which the unidimensional factor model fits the data.

By implementing the Bayesian reliability analysis in JASP we offer practitioners a low-threshold entrance to Bayesian parameter estimation and a concrete alternative to the near-universal “Cronbach’s *α*, point estimate only” approach. We hope that our work will stimulate researchers to consider reliability estimates beyond Cronbach’s *α*, and to accompany point estimates by credible intervals.

## Appendix

### Analysis in R

In order to perform the same analysis in R as we did in JASP, we need to install the R-package Bayesrel. For the latest version we use the package remotes and run the command remotes::install_github("juliuspf/Bayesrel"). We load the package, set a seed, get the data that is stored in the package, and run the full analysis by:

library(Bayesrel)set.seed(1234)# perform reliability analysis# on the ASRM-data saved in# the R-package:res <- strel(data = asrm, estimates = c("alpha", "omega"), item.dropped = TRUE,freq = FALSE)# full output summary:summary(res)

The results echo those from JASP, which is why we do not display them here.

Similar to Fig. 2, we can also calculate the posterior probability that the reliability coefficients lie in the interval [.70, .90] by:

pStrel(res, estimate = "omega", .7) -pStrel(res, estimate = "omega", .9)pStrel(res, estimate = "alpha", .7) -pStrel(res, estimate = "alpha", .9)

The convergence statistics require the coda package (Plummer et al., 2006):

library(coda)samp <- res[["Bayes"]][["samp"]][["Bayes_omega"]]# restructuring the chains# for traceplot:samp_list <- as.mcmc.list(lapply(as.data.frame(t(samp)), mcmc))traceplot(samp_list)# for R-hat or potential# scale reduction factor:gelman.diag(samp_list)$psrf[, 1]

The fit of the unidimensional model may be examined by calling

omegaFit(res, data = asrm)

## Footnotes

1 The software discussed in this paper uses the highest posterior density (HPD) interval, that is, the shortest interval that covers 95% of the posterior mass (e.g., Kruschke, 2015, chapter 4).

2 The R-package Bayesrel can be downloaded from CRAN or, for the latest version, https://github.com/juliuspf/Bayesrel. JASP can be downloaded from https://jasp-stats.org/download. The full functionality as described in this tutorial is available from JASP version 0.16.1 onwards.

3 Strictly speaking, the Bayesian reliability methods are developed for continuous multivariate normal data, but we treat the ASRM example, which is Likert-scaled, as quasi-continuous – a common practice in reliability analysis.

4 Multiple versions of McDonald’s *ω* exist, the version implemented in JASP is appropriate for unidimensional data, see McDonald (1999, Equation 6.20b). McDonald’s *ω* is computed from the parameters of a single-factor model, which – in JASP – is estimated from the centered data matrix, instead of the correlation matrix (see psych package in R).

5 To avoid varying results when rerunning the analysis, one can either increase the number of posterior samples, or set a seed in the “Convergence” tab. Users can set the seed to 1234 to obtain precisely the values we report here.

6 Common MCMC methods include Gibbs sampling (Geman & Geman, 1984), the Metropolis-Hastings algorithm (Hastings, 1970; Metropolis et al., 1953), and Hamiltonian Monte Carlo (Betancourt, 2017)

7 Contrary to common misconception the other coefficients including Cronbach’s *α* do not assume data to be unidimensional. We elaborate on this in the next section.

8 To obtain the PPC in Fig. 6, we sample the parameters from a unidimensional factor model, loadings and residuals, which we also need to compute *ω*. We combine the posterior samples of the loadings and residuals to construct a posterior sample of the model implied covariance matrix. We generate random multivariate normal data sets of the same size as the original data set with means of zero and the posterior model implied covariance matrices. We compute the eigenvalues of each of the generated data sets obtaining an empirical distribution for each eigenvalue. We plot the 2.5% and 97.5% quantiles of each eigenvalue sample as grey bars together with the eigenvalues of the original data set as black dots.

9 If data are multidimensional, researchers may divide their scale into unidimensional subscales and estimate reliability coefficients for each subscale.
